# Infectious disease management in primary care: perceptions of GPs

**DOI:** 10.1186/1471-2296-12-1

**Published:** 2011-01-11

**Authors:** Ingeborg Björkman, Mats Erntell, Marta Röing, Cecilia Stålsby Lundborg

**Affiliations:** 1 Nordic School of Public Health, Box 121 33, SE 402 42 Göteborg, Sweden; 2 Department of Public Health and Caring Sciences, Health Service Research, Uppsala Universitet, Box 564, SE 751 22 Uppsala, Sweden; 3 Department of Communicable Disease Control, County Hospital, SE 301 85 Halmstad, Sweden; 4 Division of Global Health, IHCAR, Department of Public Health Sciences, Karolinska Institutet, SE 171 77 Stockholm, Sweden

## Abstract

**Background:**

It is important to keep the level of antibiotic prescribing low to contain the development of resistant bacteria. This study was conducted to reveal new knowledge about how GPs think in relation to the prescribing of antibiotics - knowledge that could be used in efforts toward rational treatment of infectious diseases in primary care. The aim was to explore and describe the variations in GPs' perceptions of infectious disease management, with special reference to antibiotic prescribing.

**Methods:**

Twenty GPs working at primary care centres in a county in south-west Sweden were purposively selected based on the strategy of including GPs with different kinds of experience. The GPs were interviewed and perceptions among GPs were analysed by a phenomenographic approach.

**Results:**

Five qualitatively different perceptions of infectious disease management were identified. They were: (A) the GP must help the patient to achieve health and well-being; (B) the management must meet the GP's perceived personal, professional and organisational demands; (C) restrictive antibiotic prescribing is time-consuming; (D) restrictive antibiotic prescribing can protect the effectiveness of antibiotics; and (E) patients benefit personally from restrictive antibiotic prescribing.

**Conclusions:**

Restrictive antibiotic prescribing was considered important in two perceptions, was not an issue as such in two others, and was considered in one perception although the actual prescribing was greatly influenced by the interaction between patient and GP. Accordingly, to encourage restrictive antibiotic prescribing several aspects must be addressed. Furthermore, different GPs need various kinds of support. Infectious disease management in primary care is complex and time-consuming, which must be acknowledged in healthcare organisation and planning.

## Background

It has been demonstrated that the level of antibiotic use correlates with the level of antibiotic resistance [1-6]. Accordingly, it is important to keep the level of antibiotic prescribing low in order to contain the development of resistant bacteria.

Antibiotic prescribing in primary care in Sweden decreased continuously between the early 1990s and 2004, when the trend was broken and usage unexpectedly began to increase in all counties in Sweden except one [7]. No parallel increase in the occurrence of infectious diseases was registered and no other explanations were found [8]. One of the greatest increases in antibiotic prescribing compared to other counties was documented in Halland, a county in south-west Sweden. Further analysis demonstrated that the increase occurred mostly in primary care, and major variations were noted among municipalities as well as among healthcare centres (local prescription data). This interview study was initiated by the local Strama organisation (The Swedish Strategic Programme Against Antibiotic Resistance) and the local drug and therapeutics committee (DTC) to gain new understanding of antibiotic prescribing in primary care and encourage involvement in this very important question.

The aim was to explore and describe variations in GPs' perceptions of infectious disease management in primary care, with special reference to antibiotic prescribing.

## Methods

### Design and setting

A phenomenographic approach was chosen [9]. This approach has its roots in educational research but has also been used in healthcare to explore how healthcare persons view their work [10-14]. In phenomenographical studies perceptions are identified and presented as categories of description. The categories of description are based on the expressions of the respondents and are the researchers' abstractions of the perceptions in the group of respondents. The relationship among the categories of description is presented in an outcome space, if possible in hierarchical order with the most complex category on top [9,15]. A higher ranking corresponds to a higher degree of complexity, meaning that more aspects are included in the category. The focus of this study was infectious disease management in combination with restrictive antibiotic prescribing. Accordingly, the outcome space was constructed by exploring aspects relating to these issues, and categories which included aspects from both issues were regarded as more complex.

Data were collected in interviews with GPs who worked in primary care in Halland, a county in south-west Sweden.

### Participants

It has been found that 20 interviews are generally sufficient to capture the variation of perceptions [16]. This number of respondents has been used in several studies and has shown to be useful [10-14]. Accordingly 20 GPs were recruited and interviewed.

One of the authors, the chairperson of the local Strama organisation (ME), sent an email to all GPs working in primary care in the county, at that time approximately 150. The email described the purpose of the study and GPs were asked to sign up as participants. The strategy was to recruit interviewees with different kinds of experience to obtain a rich variation of views. Therefore, we looked for GPs who varied in age, sex and number of professional years, and were furthermore working at primary care centres spread geographically over the county, from both urban and rural areas, and with varying levels of antibiotic prescribing. Individual data of the level of antibiotic prescribing were not available, and instead the level of antibiotic prescribing at the medical centre where the GP worked was used. From the total of 26 volunteer GPs, 20 were chosen. Although there were not many extra GPs to choose from, the variation of predefined background characteristics was considered acceptable (see Table 1).

**Table 1:** Background characteristics of GPs*

Variable		N
*Sex*	*Women*	7
	
	*Men*	13

*Age (yrs)*	*30-39*	2
	
	*40-49*	6
	
	*50-59*	7
	
	*60-69*	5

*Years in profession*	*0-10*	3
	
	*11-20*	4
	
	*21-30*	8
	
	*31-40*	5

*Municipality*	*I*	2
	
	*II*	7
	
	*III*	4
	
	*IV*	2
	
	*V*	4
	
	*VI*	1

*Ownership of*	*Public*	10
	
*medical centre*	*Private*	10

*Level of antibiotic*	*High*	9
	
*prescribing at GP's*	*Medium*	7
	
*medical centre*	*Low*	4

*The GPs had experience from both primary care medical centres and emergency care centres.

According to the Swedish legislation ethical approval was not necessary due to the character of the study, which was a part of quality improvement activities within the county healthcare organisation. All interviewees were informed that participation was voluntary and that they could withdraw at any time, that all data would be handled confidentially and that the results from the study would be presented in a non-identifiable way.

### Data collection

All interviews were performed by the first author (IB) in March-April 2008. The interviews were held in a separate room at the GPs' primary care centres, with two exceptions. For practical reasons one participant chose to be interviewed at home and one chose a conference room in connection with a seminar arranged by the DTC.

Three main questions were used during the interviews (see Table 2). Similar questions have been used to collect information in phenomenographic studies before [11-13,17]. The questions were constructed to help the interviewees concentrate on their own experiences and to keep the focus on the phenomenon. Probing questions were asked to clarify the meaning of interviewees' statements and also bring the interview further. Interviews lasted for 40-90 minutes, were audio recorded and transcribed verbatim.

**Table 2:** Three open-ended questions were used in the interviews

Interview questions	
1	What is the core of infectious disease management?

2	Are there any problems when managing infectious diseases?

3	When is the infectious disease management successful?

Before starting the data collection three pilot interviews were performed with GPs from another county in Sweden. This led to revision of the interview questions; focus was changed from antibiotic prescribing to infectious disease management. With this change the questions were broadened to include situations in which infections were managed without antibiotics.

### Data analysis

The first author (IB) conducted the analysis and MR acted as co-reader. Both researchers have worked with several phenomenographical studies. The analysis was furthermore presented and discussed at research group meetings visited by researchers with even longer experience of this method. The analysis process included seven steps (see Table 3). The role of the co-reader was to read all the interviews, reflect on the content, and assess whether the creation of categories of description and outcome space was reasonable. In a final discussion between the analyst and the co-reader the categories and outcome space were readjusted and established.

**Table 3:** The analysing steps

Steps included in the analysing process*	
1	All interviews were read through a number of times to get an overview of the material.

2	Aspects were collected from the texts (an aspect describes critical qualities of the phenomenon, *what *it is about and *how *this is shaped).

3	The aspects were reflected on, comparing similarities and differences.

4	Preliminary categories were formed and described.

5	Aspects and descriptions were reflected on, category descriptions adjusted and a preliminary outcome space created.

6	The analysis was discussed with the co-reader.

7	Categories of description and outcome space were established together with the co-reader

*During the phenomenographic analysis the following question was kept in mind: "What does this tell me about how this GP manages patients who present with symptoms of infectious diseases?" Additionally, to focus on antibiotic prescribing, another question was kept in mind: "What does this tell me about how this GP prescribes antibiotics?"

## Results

### Five different perceptions

Five qualitatively different perceptions, A-E, were identified. The perceptions are presented below, and illustrated by quotations in Table 4. It can be noted that a common understanding concerning patients was that most patients expected their infections to be treated with antibiotics.

**Table 4:** Quotations

Perception	Quotations illustrating statements on which categories of description were based
*A. The GP must help the patient to achieve health and well-being*	"The risk of complications is an important reason for starting treatment [with antibiotics]. But I also think it is reasonable to give treatment if the course of the illness is easier." *GP:P *"They are young parents and they are a little, well, they don't have much patience, maybe they're exhausted... you feel a bit more for them in a way, almost that I think I must do everything I can to make it [the infection] pass as quickly as possible or to make it as easy as possible." *GP:N*

*B. The management must meet the GP's perceived personal, professional, and organisational demands*	"Maybe it's like this, that you have a wish to satisfy people. It's possible that's what it is, that it's more this discomfort you don't want to have." *GP:B *"As a physician it's wise to be passive, to wait-and-see. This is actually a useful way to work, as we do a lot in primary care, much more maybe than in some other specialities. But, at the same time, that may make you feel uncomfortable." *GP:G *"The face you show also creates a good client, a patient who stays with the medical centre, and this is also a way to make the finances work out. ... So, you should be very careful to take good care of your patients." *GP:C*

*C. Restrictive antibiotic prescribing is time-consuming*	"Then it's like this; many times it is much easier to prescribe antibiotics than not to. [...] you don't need any discussion, you don't need to explain yourself." *GP:A *"But to try to persuade or convince the patient that this is nothing you should treat with antibiotics, if they have expectations of getting something, is a challenge. Often you are short of time and at the same time you want the patient to be satisfied and feel listened to and confirmed." *GP:R*

*D. Restrictive antibiotic prescribing can protect the effectiveness of antibiotics*	"And often, when you explain that the guidelines are what they are and that we're trying to be cautious, to make antibiotics effective in the future, many [patients will] buy this, especially when you say that it's possible to call back and get a follow-up." *GP:D *"But then you must be able to contain this uncertainty, that is to say, it's like that. This is incorporated in my profession that I, I cannot x-ray all of them, totally safeguard ... I think you must have a larger safety margin when you are younger, when you haven't seen so much." *GP:I*

*E. Patients benefit personally from restrictive antibiotic prescribing*	"I have this attitude then, I think and believe that the body can manage itself. It will recover better and we have also seen that the immune system will not be improved by antibiotics; on the contrary you become weaker." *GP:E *"They [the patients] are known here, and they know the medical centre. And so I think it has changed a lot. When I was new in this profession I think it was more difficult to motivate, but now I think they are so well-informed." *GP:Q*

#### A. The GP must help the patient to achieve health and well-being

This perception included the concept that the patient comes to the doctor and asks for help and the role of the physician is to help the patient to achieve health and well-being. An important goal was that the patient must feel safe with the care and agree to the management. Prescribing antibiotics or not was not an issue as such. It was explained that patients often came early in the illness process, with diffuse symptoms. A few patients with infectious disease symptoms develop infections that are potentially life-threatening, and these patients must certainly be found and treated. Furthermore, it was said that patients with less serious infections also benefit from antibiotic treatment.

#### B. The management must meet the GP's perceived personal, professional, and organisational demands

The perception in B was that in general it was easier to prescribe antibiotics than to refrain. Not to prescribe antibiotics was connected with several personal and professional disadvantages including uncomfortable feelings of not satisfying the patient (who always expects antibiotics), being a passive doctor (who is not acting), risking non-successful management of the infection (which could negatively affect the patient's trust in the physician as well as the self-confidence of the physician), or being reported to the Swedish National Medical Responsibility Board. Prescribing antibiotics had organisational advantages in terms of an effective care. The consultation usually ended quicker; more patients were treated and time was saved for other patients with more complicated diseases. When patients could not be followed up antibiotics were prescribed more frequently in order to keep control.

However, in contrast to the above, there were also GPs expressing that it was the restrictive antibiotic prescribing that met their personal and professional demands.

#### C. Restrictive antibiotic prescribing is time-consuming

In this perception there was an awareness of restrictive antibiotic prescribing and that this should be considered. However, it was said that due to the general public's ignorance about symptoms and treatment of common infections there was a constant discussion with patients on infectious disease management. It was explained that primary care infections were often medically uncomplicated but nevertheless the encounter between GP and patient took a long time due to the need for discussion and education. Similar discussions were also held with professionals working at children's day-care centres or professionals working at old age homes or relatives of the elderly. An exception was parents of children with otitis media who had adopted the perception that these infections can be cured without antibiotics and thus a discussion was often easier. It was said that this was a result of previous information campaigns directed at the public.

#### D. Restrictive antibiotic prescribing can protect the effectiveness of antibiotics

Here it was said that restrictive antibiotic prescribing must be practiced in order to protect the effectiveness of antibiotics. Not being able to treat infections in the future was considered a real threat. Of course some patients need antibiotics, but the most common infections can be cured without antibiotics. One or two days of extra suffering may not be convenient for the patient, but is not dangerous. Occasionally, a patient will develop a serious infection that probably could have been avoided if antibiotics had been prescribed at the first meeting. This is however something both patients and physicians must get used to. It was said that such situations were managed by instructing patients to observe their symptoms and contact the GP again if they got worse. An effect of a new healthcare policy in the county was that now it was easy for patients to contact their GPs.

#### E. Patients benefit personally from restrictive antibiotic prescribing

The perception in E has many similarities with perception D including the notion that antibiotics must be protected for the future. However, in this perception it was furthermore stated that the patients benefit personally from restrictive antibiotic prescribing. When the body cures the infection without antibiotics the patient's immune system is not negatively affected, as it is when antibiotics are used. As in perception D, patients were instructed to contact the GP again if symptoms worsened. Some GPs who expressed perception E said that their patients did not want antibiotics and just came to be assessed to make sure that antibiotics were not needed. These patients were said to be the GPs' "own" patients who had adopted the same attitude towards infectious disease management as the GP.

### The outcome space

**Figure 1:**
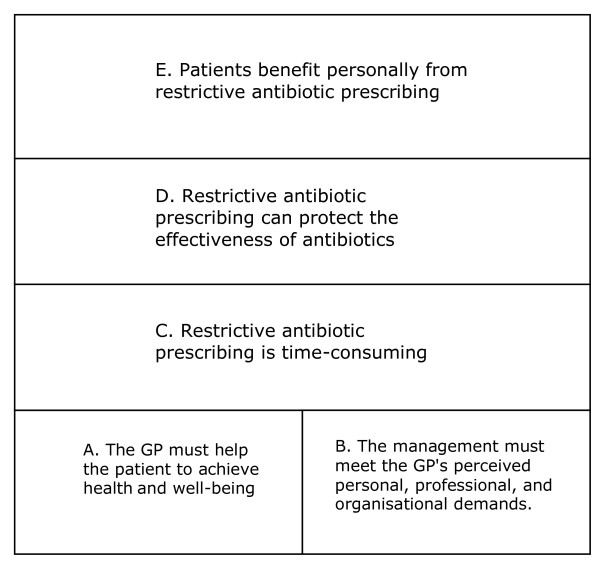
**The outcome space**. The outcome space describes the relationships among the categories of description. Here categories are presented in a hierarchical order at four levels. Dominating aspects in the five categories of description were: A) the health and well-being of the patient; the duty of the GP to help patients, B) the GP's personal and professional experiences and expectations; perceived organizational demands, C) restrictive antibiotic prescribing should be considered; the general public is ignorant of common infectious diseases; restrictive antibiotic practice is time-consuming; D) the risk of non-effective antibiotics in the future is a reality; antibiotic use leads to resistant bacteria; patients and GPs must stand some discomfort for the sake of the future, and E) antibiotics have impact on patients' immunological resistance; patients with common infections who refrain from antibiotics are strengthened in the long run.

The relationship among categories of description is presented in the outcome space (Figure 1). Here a category placed at a higher level corresponds to an increased likelihood of restrictive antibiotic prescribing in practice. At the first level restrictive antibiotic prescribing was not an issue. At the next level restrictive prescribing was considered but the result depended on what happened in the interaction between patient and physician. At the two highest levels, restrictive antibiotic prescription was important and was practiced.

## Discussion

This study suggests that different GPs perceive infectious disease management in primary care in different ways, and also vary in their perceptions of the role of antibiotics in disease management. Two perceptions (D and E) were associated with a strong focus on restrictive antibiotic prescribing, whereas the others were not (A, B, and C). This means that GPs who hold perceptions similar to D and E are more likely to apply restrictive antibiotic prescribing when they meet primary care patients with infectious disease symptoms.

Our findings both support previous studies and give new information. One novelty lies in the descriptions of our findings, as a variation of perceptions among GPs. Perceptions are, according to phenomenographic theory, intertwined with the persons' lived experiences and actions [9], which means that they tell something about the participants' experience and what actions they take. Variations in ways of prescribing antibiotics have also been described as a result of different physicians' characters or behaviour [18-20]. Our findings demonstrate that physicians have different perceptions of infection management, which probably reflect different ways of prescribing antibiotics. Thus, because physicians have different perceptions they probably need different kinds of support in the promotion of restrictive antibiotic prescribing. Furthermore we describe perceptions that favour restrictive antibiotic prescribing whereas many studies concentrate on obstacles to restrictive prescribing.

Previous studies have explained that GPs must consider many factors besides the risk of antibiotic resistance, including the immediate duty to the patient, cost, patient pressure and legal issues [21-24]. For instance, prioritizing the patient's immediate needs may be a reason for choosing a broad-spectrum antibiotic [25]. Organisational impact has also been described; the likelihood of antibiotic prescribing increases with lack of continuity of medical care [20], and with work under pressure [26]. All these factors could be identified among the physicians in our study, but were here more in the foreground in some perceptions and more in the background in others.

Two recent studies used phenomenographic approaches to study perceptions of medical prescribing among physicians. One focused on antibiotic prescribing at hospitals [27]. A major difference between that study and the present one was that the perceptions of antibiotic prescribing in hospital care were not influenced by patient-physician interaction, whereas this was a factor in most of the perceptions in the present study. The other study focused on perceptions of medical prescribing (not specifically antibiotics) among GPs [28]. Five categories of description were found and the authors conclude that the most influential factor was the physician's patient relation approach. Patient-physician interaction may naturally be more important in primary care, as patients play a more active role in their treatment.

It was perceived that it was easier to prescribe antibiotics than to refrain, and one reason was that this would always satisfy the patient. Antibiotic prescribing as a method of satisfying patients has been documented before [29-31]. Documentation supports the idea of patient expectations of antibiotics [32-34]. However, it is possible that patient pressure and expectations are not real, but constructed in the minds of the physicians [35,36]. A Swedish study demonstrates that patients reported higher confidence in physicians who had given information to them, whereas whether they had been given antibiotics or not was less important [37]. A Scottish study reveals that physicians felt pressed by patients to prescribe antibiotics, but patients said they were willing to wait to confirm whether antibiotics were justified or not [38].

Thus, it seems important not to take patients' expectations for granted. It is probably advantageous to involve patients in actions to prevent antibiotic resistance. When they are asked, patients express concern about antibiotic resistance and say they want to cooperate to decrease the problems [34,38]. Material that facilitates the meeting with the patient can be helpful [39]. The general public also needs to know about the relation between antibiotic use and resistant bacteria. People do not believe they have roles in antibiotic resistance and do not understand that they can contribute by asking less often for antibiotics for minor infections [40]. The GPs in our study perceived that many patients were ignorant of common symptoms and did not know how to treat themselves. Information activities directed at patients as well as promotion in mass media have been suggested [41]. A review concluded that antibiotic use was reduced by such campaigns, at least when the public and the physicians were targeted simultaneously [42]. In our study the GPs mentioned that parents had learned from information campaigns that otitis media in children can be cured without antibiotics.

However, in spite of the common notion among GPs that patients generally expected antibiotics, we found two perceptions in which GPs said they practiced restrictive prescribing (D and E). A major difference in these perceptions was that physicians perceived that the effect of antibiotics was a real threat and furthermore said that they had experienced that it was possible, or even beneficial, to refrain from antibiotics in most common infections. It was also said here that restrictive antibiotic prescribing was time-consuming (as in perception C), but discussions were considered necessary for the practice, not a hindrance. It has been shown that GPs can increase their communication ability in patient encounters without prolonging the counselling time [43]. However, in our study most patient-physician encounters were thought of as time-consuming, except for encounters with parents of children with otitis media, as discussed above.

In one of these perceptions (perception D) we traced a conflict between the desire to save the effectiveness of antibiotics for the future and the basic concept of helping patients who suffer (perception A). This conflict was not present in perception E, where antibiotics were also to be saved for the future. Here restrictive antibiotic use moreover benefited the patients of today, who then did not have to be negatively affected by the antibiotic. Such a perception may aid GPs in following restrictive antibiotic prescription recommendations.

The perception that restrictive antibiotic prescribing is time-consuming (C) contains an important message for the organisation and planning of healthcare. Primary healthcare policies supporting restrictive antibiotic prescription must allocate resources to give physicians room for discussions with patients. Infectious diseases in primary care may look simple from a biomedical point of view. However, from a psychosocial point of view they are complicated and complex. This understanding seems to be underestimated and not considered in healthcare today.

Besides time allocation and patient involvement, some GPs must adopt new ideas to consider restrictive antibiotic prescribing in infectious disease management. For instance, GPs must believe that their actions make a difference. Documentation shows that physicians sometimes regard themselves as not being a part of the problem, and think that antibiotic resistance is a national problem in which they are not involved [31,38,44]. However, a relationship has also been seen between use of antibiotics and antibiotic resistance at an individual level [3,4,6]. For these GPs information and education on prescribing and antibiotic resistance are needed [24,41].

### Methodological considerations

Participant selection aimed at including GPs with varying experiences, in order to get a rich description of the phenomenon [45]. We aimed at recruiting GPs who were low, medium and high prescribers of antibiotics, respectively. Individual prescription data were however not available and the levels of antibiotic prescribing at the primary care centres where the GPs worked were used instead. Participation was voluntary; a possible risk was that only GPs who were already engaged in the question of restrictive antibiotic prescribing chose to participate. However, the collected material gave a rich variation, indicating that the study gives a good picture of perceptions among GPs. Similar findings in scientific literature suggest that our findings are not only applicable to GPs in Sweden but also internationally.

To ensure high quality of collected material we used interview questions that 1) were similar to questions that have been useful in previous studies [11-13,17], and 2) were constructed to help interviewees focus on their own experiences. Pilot testing resulted in revision of interview questions and strengthened the quality of the interview material. The analysis was performed systematically and carefully by a researcher with experience in phenomenographic analysis. To enhance trustworthiness, an experienced co-reader assessed the analysis.

## Conclusions

The study describes the complex nature of infectious disease management in primary care and furthermore describes that GPs have different perceptions of the management as well as the role of antibiotics. Five different perceptions were identified. In two of the perceptions restrictive antibiotic prescribing was always practiced, and in one, sometimes practiced, depending on the interaction with the patient. To encourage restrictive antibiotic prescribing interventions must address several aspects; however, different GPs need different kinds of support. Some GPs need to learn about risks of resistant bacteria and to be aware of the impact they can play on the level of antibiotic resistance; others primarily need training in communication skills. Infectious disease management in primary care is complex and time-consuming, which must be acknowledged in the healthcare organisation and planning.

## Competing interests

The authors declare that they have no competing interests.

## Authors' contributions

ME and CSL prepared the study and designed it in cooperation with IB. IB carried out the interviews and drafted the manuscript. IB and MR analysed the interviews. ME, MR, and CSL read and gave comments on the manuscript. All authors approved the final version of the manuscript.

## Pre-publication history

The pre-publication history for this paper can be accessed here:

http://www.biomedcentral.com/1471-2296/12/1/prepub
